# SIX1 Activates STAT3 Signaling to Promote the Proliferation of Thyroid Carcinoma via EYA1

**DOI:** 10.3389/fonc.2019.01450

**Published:** 2019-12-20

**Authors:** Deguang Kong, Anping Li, Yu Liu, Qiuxia Cui, Kun Wang, Dan Zhang, Jianing Tang, Yaying Du, Zhisu Liu, Gaosong Wu, Kongming Wu

**Affiliations:** ^1^Department of General Surgery, Zhongnan Hospital of Wuhan University, Wuhan, China; ^2^Department of Medical Oncology, The Affiliated Cancer Hospital of Zhengzhou University and Henan Cancer Hospital, Zhengzhou, China; ^3^Department of Geriatrics, Tongji Hospital of Tongji Medical College, Huazhong University of Science and Technology, Wuhan, China; ^4^Department of Thyroid and Breast Surgery, Zhongnan Hospital of Wuhan University, Wuhan, China; ^5^Department of Thyroid and Breast Surgery, Tongji Hospital of Tongji Medical College, Huazhong University of Science and Technology, Wuhan, China

**Keywords:** SIX1, EYA1, STAT3 signal, thyroid papillary carcinoma, tumor growth

## Abstract

As a critical member of the Retinal Determination Gene Network (RDGN), SIX1 has been regarded as a tumor promoter in various types of cancer. However, its role in papillary thyroid carcinoma (PTC) has never been investigated. In this study, thyroid carcinoma tissue microarray staining was employed to identify the expression patterns of SIX1 and its co-activator EYA1. Papillary thyroid cancer cell lines, BCPAP, and TPC-1 cells were used to investigate the potential mechanism of SIX1 *in vitro* and *in vivo*. Flow cytometry analysis, MTT assay, the growth curve assay, colony formation assay, EdU incorporation and xenograft assay were performed to demonstrate the role of SIX1 in the malignant change of PTC cells. Western blot and Real-time PCR were used to detect the interaction among the SIX1, EYA1, and STAT3 signaling. In comparison with normal tissue, high expressions of SIX1 and EYA1 were associated with a malignant tumor. Importantly, SIX1 strongly correlated with EYA1 in thyroid carcinoma tissue microarray. Functional assays indicated SIX1 increased EYA1 expression by stabilizing EYA1 at the post-transcriptional level. Besides, SIX1 promoted the proliferation and invasion of thyroid carcinoma via activation of STAT3 signaling and its downstream targets in an EYA1-dependent manner. SIX1 can integrate with EYA1 to contribute to PTC development via activation of the classical STAT3 signaling. These data suggested targeting the abnormal activation of the SIX1/EYA1 complex may represent a novel therapeutic strategy for advanced PTC patients.

## Introduction

The incidence of thyroid cancer is rapidly increasing in recent two decades, and it has become the most common endocrine malignancy worldwide (1, 2). As the predominant pathological type of thyroid carcinoma, papillary thyroid carcinoma (PTC) accounts for more than 80% of the total (3). For most of PTC patients, conventional surgical thyroidectomy with radioactive iodine ablation and thyrotropin hormone-suppressive levothyroxine can obtain a satisfactory outcome (4). However, about 10% of cases suffer from a locally advanced or metastatic disease at diagnosis, which are the most frequent causes of thyroid cancer-related death (5). Understanding the molecular basis of thyroid cancer is essential for developing effective strategies for advanced PTC patients (6).

The development of therapeutic agents that target tumor driver genes is the fundament for precision medicine (7). Exciting discoveries like the genetic duet of *BRAF* ^*V*600*E*^ and *RAS* mutations have led to significant progress in targeted therapies. Several *BRAF* tyrosine kinase inhibitors are either approved or undergoing investigation for patients with malignancies carrying *BRAF* mutations (8). However, the complexity in molecular subtypes of PTCs is still a challenge in exploring novel biomarker for guiding personal treatment in advanced stage (9). Meanwhile, alterations of critical signaling pathways in thyroid development like MAPK and PI3K-AKT can initiate tumorigenesis and promote tumor metastasis (10). Targeting the aberrant expression of development related genes or signaling may provide an opportunity for the molecular-based treatment of thyroid cancer.

In this respect, the *retinal determination gene network* (RDGN) family is required for organismal development in mammalian (11). This regulatory network mainly consists of *dachshund* (*dac*/Dach), the Six family transcription factor *sine oculis* (*so*/Six) and a tyrosine phosphatase *eyes absent* (*eya*/Eya). The balance of Dach/Eya/Six network governs the tissue differentiation (11). Recently, the abnormal activation SIX1 and EYA family have been proved to be involved in the development of multiple cancers. SIX1 promoted malignant transformation of mammary epithelial cells, including increased proliferation and anchorage independent growth by activating cyclin A1 (12). Via activation of TGF-β and MAPK signal, SIX1 enhanced the accumulation of cancer stem cells (CSCs) as well as induced epithelial-mesenchymal transition (EMT), and even switched the role of TGF-β/SMAD signal from tumor suppressors to oncogenic proteins in breast cancer (13–16). SIX1 can inhibit p53 by upregulating microRNA-27a-3p and downregulating ribosomal protein L26 (RPL26) to diminish the p53-mediated tumor suppression across different cancer types (7). Most recent study found the microRNA-548a-3p/SIX1 axis strongly linked aerobic glycolysis to carcinogenesis (17). SIX1 and EYA can integrate with other signal pathways to modulate the apoptosis, cell proliferation and tumor growth (18). To date, the profiles of SIX1 and EYA1 have been independently identified as a prognostic biomarker in breast cancer (13, 19). However, the functional relationship of SIX1 and EYA1 in thyroid cancer remains to be discovered.

Abnormal re-activation of embryological genes can trigger the tumorigenesis. A previous study has indicated that the Eya1 and Six1 were required for the morphogenesis of mammalian thyroid (20). Given these observations, the role of SIX1 and EYA1 in tumorigenesis and progression of PTC deserves further investigation. In this study, we explored the role of SIX1 in PTC through *in vitro* and *in vivo* experiments, which implicated SIX1 coordinated with EYA1 to drive neoplastic growth and invasion via activation of the classical STAT3 signaling.

## Materials and Methods

### PTC Tissue Microarray and Immunohistochemistry

Commercially available tissue microarray (TMA) slides (TH8010 and TH802a, US Biomax, Inc.) were purchased for immunohistochemistry (IHC) analysis. Specific primary antibodies against SIX1 (Sigma, USA) and EYA1 (Proteintech, China) were used for IHC with a 2-step protocol (21). Whole slide image capture was performed on the EVOS auto cell image system (Life technology, USA). For semi-quantitative evaluation of protein level in tissue, the staining intensity was graded as previously described (22). The immunohistochemical score were assessed by two experienced pathologists without knowledge of patients' characteristics. Scores were calculated on intensity and percentage of positive staining tumor cell nuclei or cytoplasm in the whole tissue stains were evaluated according to Fromowitz Standard. Briefly, the staining intensity was graded as follows: no staining, 0; weakly positive, 1; moderately positive, 2; and strongly positive, 3. The percentage of positive cells was into four grades: 0–25% staining, 1; 26–50% staining, 2; 51–75% staining, 3; and 76–100% staining, 4. The multiplication of the intensity and percentage scores was used to calculate the final staining score. For quantification, all stains were assessed at 200× magnifications and at least three fields from each core were counted.

### Cells Culture and Transfection

Human papillary thyroid cancer cell lines BCPAP, NPA and TPC-1 were provided by Dr. Du (Tongji Hospital of Tongji Medical College) and cultured in recommended condition. Cell line authenticity was confirmed by Short Tandem Repeat (STR) DNA profiling (the STR profiles were shown in Supplementary Materials). All experiments were performed using cell lines from passage 6 to 25. Cells were seeded at 50% confluence in 6 cm plate on the day before transduction. HEK 293T cells were transfected with pLV vector or pLV-SIX1 expression plasmid with package plasmids by Lipofectamine™ 2000 (Invitrogen, Carlsbad CA, USA). The supernatant was collected and polybrene (1:1,000) was added into the supernatant. The mixed supernatant was applied to recipient cells for infection. The expression of SIX1 was verified by quantitative reverse transcription-PCR (qRT-PCR) and Western blot. For small interfering RNA (siRNA)-mediated downregulation of EYA1, BCPAP-Vector and BCPAP-SIX1 cells were seeded in 6-well plates and transfected with siRNA or scramble control (Ribobio Company, Guangzhou, China) duplexes using Lipofectamine™ 2000 (Invitrogen, Carlsbad CA, USA). EYA1 siRNA: (sense) 5′-CAGGAAAUAAUUCACUCACAAdTdT-3′; (antisense) 5′-UUGUGAGUGAAUUAUUUCCUGdTdT- 3′.

### Western Blot Analysis

Cell and tissue lysates were extracted using ice-cold RIPA buffer and measured using a bicinchoninic acid (BCA) protein assay kit (Promoter, China). Proteins were resolved on 10% SDS-polyacrylamide gels and transferred to PVDF membranes. The antibodies used in Western blot were as follows: SIX1, EYA1, C-MYC (Santa Cruz, USA), STAT3 (Cell Signaling Technology, USA), p-STAT3 (Tyr705) (Cell Signaling Technology, USA), BCL-XL (Cell Signaling Technology, USA), Caspase 3 (Proteintech, China), Cleaved PARP1 (Ruiying Bio, China), Cleaved Caspase 9 (Ruiying Bio, China), β-tublin (Cell Signaling Technology, USA), GAPDH (Cell Signaling Technology, USA) and VINCULIN (Sigma, USA).

### Quantitative Reverse Transcription-PCR (qRT-PCR)

RNA was prepared from PTC cells with the TRIzol reagent (Invitrogen, USA). cDNA was reversed from 1 μg total RNA using a reverse transcription kit (TOYOBO, Japan). RT-qPCR was performed with the SYBR^®^ Green Real-time PCR Master Mix Kit (TOYOBO, Japan). Gene expression was normalized to GAPDH. The primer sequences for real-time RT-PCR were as follows: SIX1: (forward) 5′-ACAAGAACGAGAGCGTACTCA-3′, (reverse) 5′-CTCCACGTAATGCGCCTTCA-3′; EYA1: (forward) 5′-GTTCATCTGGGACTTGGA-3′, (reverse) 5′-GCTTAGGTCCTGTCCGTT-3′; GAPDH: (forward) 5′-CAATGACCCCTTCATTGACC-3′, (reverse) 5′-GATCTCGCTCCTGGAAGATG-3′.

### Cell Proliferation Assays

For MTT assay, 2,000 cells were seeded into 96-well plates and analyzed by adding MTT (tetrazolium bromide, 5 mg/mL, GE Healthcare) as previously described (22). To measure the growth curve, 2.5 × 10^3^ cells were seeded in 24-well culture plates and the numbers of viable cells were serially counted for 6–7 days. Colony formation assay was performed as previously described (23). Two weeks later, cells were fixed with 4% paraformaldehyde and stained by 0.5% crystal violet for visualization and counting on the plate.

### Cell-Cycle Analysis

About 3 × 10^5^ cells were seeded into a 6 cm Petri dish. After 24 h incubation, the cells were collected and fixed with 75% cold ethanol at −20°C overnight. DNA was incubated with 200 μL RNase A (1 mg/mL) and 500 μL propidium iodide (PI, 100 μg/mL) for 30 min at room temperature in the dark and analyzed by using the FACSort flow cytometer (Becton, Dickinson Company, USA). The data were analyzed with ModFit LT V2.0 software (Becton, Dickinson Company, USA).

### Transwell Migration and Invasion Assay

Transwell chambers (pore size 8.0 μm) (Corning Inc., USA) were either uncoated (migration assay) or coated (invasion assay) with Matrigel as previously described (23). All experiments were conducted in triplicate.

### Annexin V-FITC/PI Assay

The apoptosis was determined by the Annexin V-FITC/PI apoptosis detection kit (BD Biosciences, USA). Briefly, cells were treated with 200 μM H_2_O_2_ (Sigma, USA) for 24 h. The cells were collected, and then resuspended in 200 μL of binding buffer. After incubation of Annexin V-FITC and PI for 15 min at room temperature, 300 μL binding buffer was added to the cells and the results were analyzed by flow cytometry (Beckman-Coulter Inc., USA). The experiment was repeated in triplicate.

### Ethynyl-20-deoxyuridine (EdU) Incorporation Assay

EdU incorporation assay was performed with EdU assay kit (Ribobio, China). Briefly, 1 × 10^3^ cells per well were culture in 96-well plates for 48 h, and then 50 μM of EdU was added to each well and cultured for additional 2 h. The cells were fixed with 4% formaldehyde for 15 min and treated with 0.5% Triton X-100 for 20 min. After washing with PBS, 100 μl of 1 × Apollo reaction cocktail was added and incubated for 30 min. After staining with 100 μl of Hoechst 33342 for 30 min, the cells were visualized under EVOS cell image system. The results were analyzed by Image-Pro Plus 6.0 software (Media Cybernetics, USA). All experiments were done in triplicate and three independent repeating experiments were performed.

### Immunofluorescent Labeling of SIX1 and EYA1

Cells were seeded in a 24-well plate (5 × 10^4^cells/well) and cultured for 48 h, then fixed with 4% paraformaldehyde. Next, cells were permeabilized with 0.1% Triton X-100 and blocked using 5% goat serum for 30 min. Cells were further incubated overnight with primary antibodies against SIX1 (anti-mouse, 1:100, Santa Cruz, USA), EYA1 (anti-rabbit, 1:100, Proteintech, China). Next day, cells were incubated with either Alexa Fluor 594 or Alexa Fluor 488 for 1 h. Nuclei were visualized with DAPI stain. The stained cells were examined with EVOS cell image system.

### Tumor Formation Assay

Five- to 6-week-old female NOD/SCID mice were purchased from Beijing HFK Bioscience Limited Company and maintained under the Specific Pathogen-Free (SPF) conditions at Laboratory Animal Centre. All the protocols were reviewed and approved by the Institutional Ethics Committee and performed in accordance with national guidelines. All mice were randomly divided into two groups: TPC1-shControl (*n* = 20) and TPC1-shSIX1 (*n* = 20). Cells were counted and serially diluted in 100 ml of 1:1 PBS/Matrigel, and injected subcutaneously. The mice were monitored every 3 days. At 3 weeks post-injection, all mice were euthanized and analyzed for tumor formation. Tumors were collected for further Western Blot analysis.

### Statistical Analysis

GraphPad Prism 6.0 software was used for the statistical analyses. The IHC scores were tested whether the data matched normal distribution or not. If it was, then the difference between groups were conducted by using parametric statistics (*Student t*-test), otherwise performing non-parametric statistics (*Mann-Whitney* test). The correlations between clinicopathological and immunohistochemical variables were calculated according to *Person* χ^2^-test. Cell culture experiments differences between the groups were evaluated by the *Student t*-test, including EdU assays, MTT activity, growth curve assays, colony formation assay, Transwell migration, and invasion assays, Annexin V-FITC/PI assays, PCR and western blot assays. All data were expressed as mean ± standard error. *P* < 0.05 were considered statistically significant.

## Results

### Both SIX1 and EYA1 Were Increased in PTC Patients

**Figure 1:**
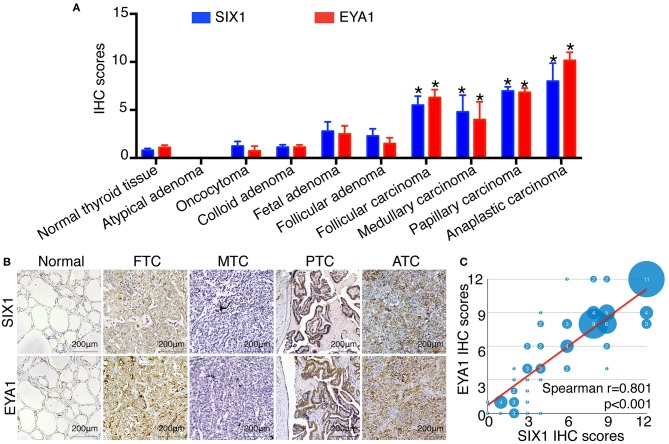
The expression of SIX1 and EYA1 in PTC tissues. The semi quantitative results displayed as median ± stand error **(A)**. The relative expression of SIX1 and EYA1 in thyroid carcinoma tissue microarray, including normal thyroid tissue, atypical adenoma, oncocytoma, colloid adenoma, fetal adenoma, follicular adenoma, follicular carcinoma, medullary carcinoma, papillary carcinoma, and anaplastic carcinoma, ^*^represents a statistical difference was found compared with normal tissue. **(B)** Representative immunohistochemistry images of SIX1 and EYA1 expression in normal thyroid, papillary thyroid carcinoma (PTC), follicular thyroid carcinoma (FTC), medullary thyroid carcinoma (MTC), and anaplastic thyroid cancer (ATC). **(C)** The positive relationship between SIX1 and EYA1 in thyroid malignant carcinoma.

To identify the expression pattern of SIX1 and EYA1 in human thyroid tissue as well as tumor, we performed IHC analysis by using two tissue microarrays (TH8010 and TH802a). After excluding the duplicated reports (*n* = 6) and detached sample (*n* = 9), two tissue microarrays contained 145 samples, including normal thyroid tissue (*n* = 10), atypical adenoma (*n* = 1), oncocytoma (*n* = 4), colloid adenoma (*n* = 15), fetal adenoma (*n* = 10), follicular adenoma (*n* = 10), follicular carcinoma (FTC, *n* = 18), medullary carcinoma (MTC, *n* = 5), papillary carcinoma (PTC, *n* = 66), and anaplastic carcinoma (ATC, *n* = 6). The IHC score for each type was shown in Figure 1A. In comparison with normal tissue, SIX1 and EYA1 kept low expression in benign tumor and no statistic difference was found for either SIX1 or EYA1 between groups. However, a significant increase in SIX1 and EYA1 was detected in malignant tumor, including FTC (*p* = 0.011, *p* = 0.038; respectively), MTC (*p* = 0.006, *p* = 0.043; respectively), PTC (*p* < 0.001, *p* < 0.001; respectively) and ATC samples (*p* = 0.002, *p* < 0.001; respectively) (Figure 1A). The representative images of IHC staining for normal and cancerous tissue were shown in Figure 1B. Intriguingly, SIX1 was tightly linked to EYA1 in malignant tumor. The analysis from Spearman correlation based on all malignant sample in these tissue microarrays indicated that SIX1 had a strongly positive relationship with EYA1 (Figure 1C). BRAF mutation is the most common mutation in PTC (8). To test whether there was relationship between SIX1/EYA1 and BRAF^*V*600*E*^ mutation, the GEO database (GSE54958) was applied to analyze. There was no difference in the expression of SIX1/EYA1 and BRAF^*V*600*E*^ mutation (Data not shown).

Since PTC is the most common type of thyroid carcinoma and also occupied the majority of the tissue microarrays in this study, we next examined the association of SIX1 and EYA1 expression with clinicopathological parameters. Based on the semi-quantitative criteria, we defined IHC score over 6 (>6) was higher and scores under 6 (≤6) was lower expression. We found that the levels of SIX1 and EYA1 expression were significantly associated with age, lymph node metastasis (two sample missed this information, tissue ID: Etg030430 and Etg030007) and clinical stage, but not tumor size (Table 1). Based on these data, high expressions of SIX1 and EYA1 are closely correlated with thyroid malignant tumor. SIX1 may cooperate with EYA1 and play a key role in lymph node metastasis via a BRAF-independent manner.

**Table 1:** Association between SIX1/EYA1 expression and clinicopathologic factors in papillary carcinoma (TH8010 + TH802a).

**Characteristics**	**SIX1 expression**			**EYA1 expression**		
	**Low (≤6)**	**High (>6)**	***p***	**Low (≤6)**	**High (>6)**	***p***
Sex			0.481^*^			0.177^*^
Male	4	8		3	9	
Female	24	30		25	29	
Age (y)	38.96 ± 2.429	49.63 ± 2.662	0.006^†^	39.5 ± 2.434	49.24 ± 2.7	0.012^†^
Tumor size			0.347^*^			0.145^*^
≤4 cm (T1 + T2)	12	12		13	11	
>4 cm (T3 + T4)	16	26		15	27	
Lymph node metastasis			0.043^*^			0.030^*^
Absent	23	23		24	22	
Present	4	14		4	14	
Stage			0.015^*^			0.015^*^
I + II	24	22		24	22	
III + IVA	4	16		4	16	

* *Person chi-square test*.

† *Independent-sample t-test*.

### SIX1 Increased the Proportion of Cells in S Phase in PTC Cell Lines

**Figure 2:**
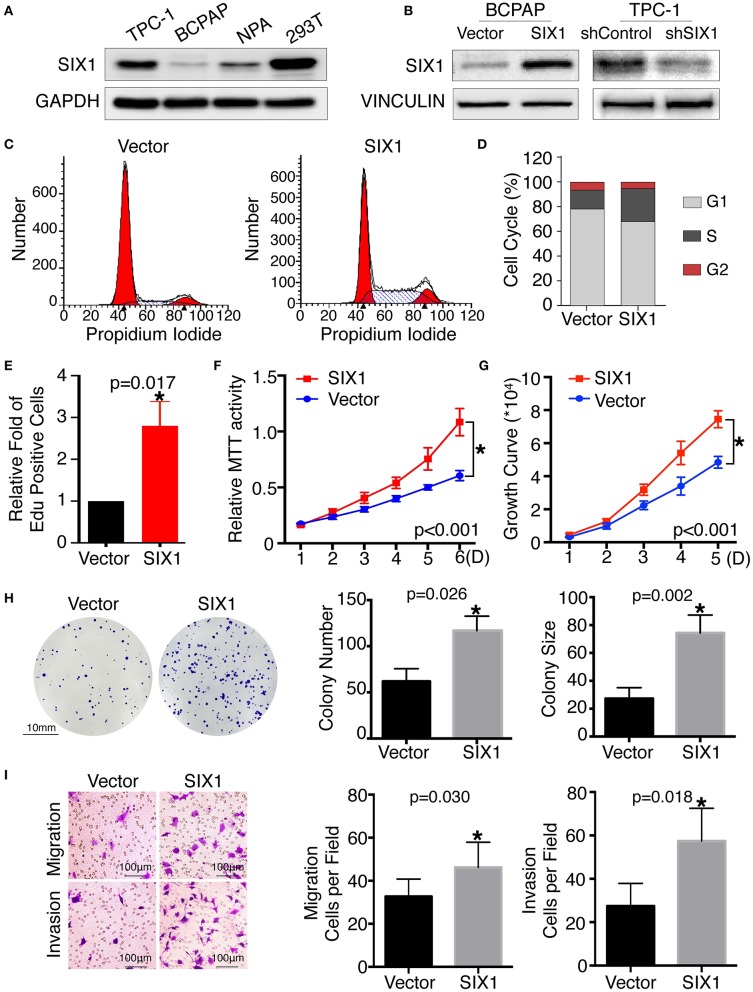
Ectopic expression of SIX1 increased the proportion of cells in S phase and promoted proliferation, migration and invasion in BCPAP cell lines. Protein abundance of SIX1 in PTC cell lines by western blot analysis **(A)**. Western blot analysis confirmed the efficiency of ectopic expressing SIX1 in BCPAP cells or silencing SIX1 in TPC-1 cells **(B)**. Representative images of cell cycle plot **(C)** and quantitative results (*n* = 3) **(D)**. Quantitative results of EdU staining of cells with SIX1 overexpression (*n* = 3) **(E)**. MTT assays of BCPAP cells with ectopic expression of SIX1 (*n* = 5) **(F)**. Growth curve assays of BCPAP cells with ectopic expression of SIX1 (*n* = 5) **(G)**. Representative images of colony formation assays of BCPAP cells with ectopic expression of SIX1 and quantitative results (*n* = 3) **(H)**. The representative images of migration and invasion assay in BCPAP cells with SIX1 overexpression and quantitative analysis (*n* = 3) **(I)**. ^*^p < 0.05.

Since SIX1 is a DNA-specific transcriptional factor, whereas EYA1 basically acts as a co-factor to increase SIX1-dependent function (11), we mainly focused on the functional role of SIX1 in thyroid cancer for first. The base line expressions of SIX1 were detected by western blot in PTC cell lines, including BCPAP, TPC-1, and NPA, with HEK 293T cell as a positive control. The results showed that SIX1 were barely expressed in BCPAP cells, whereas highly enriched in TPC-1 cells (Figure 2A). The next step was establishing the thyroid cell lines with either overexpression or knockdown of SIX1 by employing lentivirus-mediated gene transfer system. BCPAP cells stably overexpressing SIX1 were successfully transduced by pLV-SIX1 (BCPAP-SIX1) and pLV empty vector (BCPAP-Vector). Meanwhile, lentivirus shRNA expression vector for SIX1 or scramble control were transduced into TPC-1 cells (Figure 2B).

**Figure 3:**
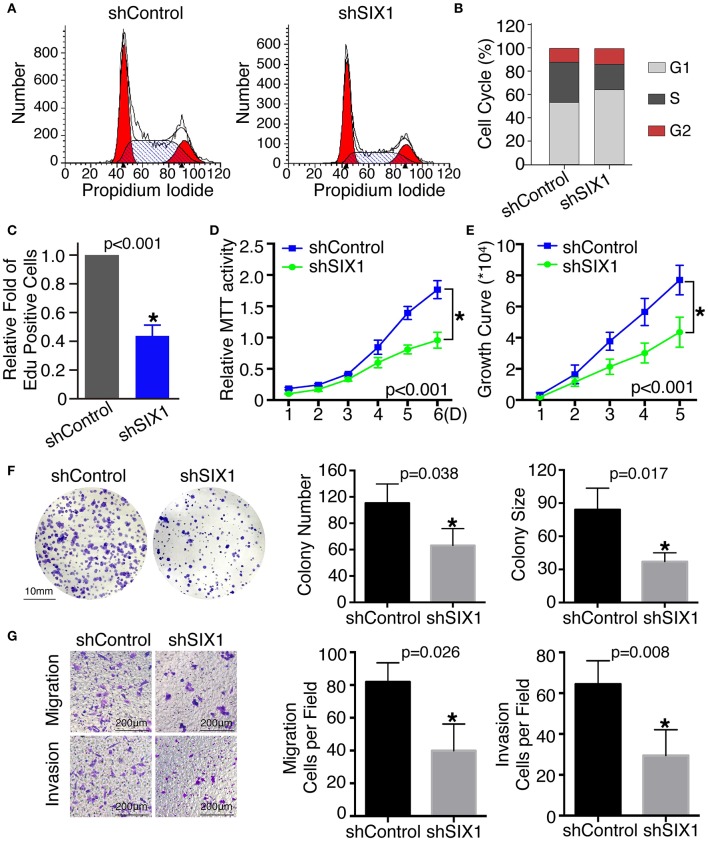
Knockdown SIX1 decreased the proportion of cells in S phase and inhibited proliferation, migration, and invasion in TPC-1 cell lines. Representative images of cell cycle plot **(A)** and quantitative results (*n* = 3) **(B)**. Quantitative results of EdU staining of cells with SIX1 knockdown (*n* = 3) **(C)**. MTT assays of TPC-1 cells after silencing SIX1 (*n* = 5) **(D)**. Growth curve assays of TPC-1 cells after silencing SIX1 (*n* = 5) **(E)**. Representative images of colony formation assays of TPC-1 cells after silencing SIX1 and quantitative results (*n* = 3) **(F)**. The representative images of migration and invasion assay in TPC-1 cells after silencing SIX1 and quantitative analysis (*n* = 3) **(G)**. ^*^p < 0.05.

To assess the role of SIX1 in cell cycle regulation, propidium iodide staining and flow cytometry were performed. Overexpression of SIX1 significantly decreased the percentage of cells in the G0/G1 phase (78.47 ± 2.37% vs. 67.31 ± 1.56%, *p* = 0.017) and increased the subpopulation in the S phases (16.33 ± 2.44% vs. 27.68 ± 1.72%, *p* = 0.019) in BCPAP cells (Figures 2C,D). Conversely, knockdown SIX1 in TPC-1 cells induced a significant increase of G0/G1 phase (53.73 ± 0.87% vs. 64.79 ± 1.46%, *p* = 0.003) and a decrease in the S-phase population (34.42 ± 0.61% vs. 22.77 ± 1.32%, *p* = 0.001) compared with control group (Figures 3A,B). To further evaluate the effect of SIX1 in cell cycle regulation, EdU incorporation assay was examined. In accordance with results of flow cytometry, the proportion of EdU positive cells was increased by 2.84-folds induced by SIX1 overexpressing in BCPAP cells (Figure 2E). And the number of EdU positive cells in TPC-1-shSIX1 group was reduced by 40% in comparison with TPC1-shControl (Figure 3C). Taken together, these data demonstrate that SIX1 promoted cell cycle progression in thyroid cancer cells.

### SIX1 Overexpression Induced Proliferation, Cell Migration, and Invasion in PTC

To examine the effect of SIX1 on PTC cellular proliferation, MTT assays, cell growth curve, and colony formation assays were performed to assess the proliferative ability. MTT assays showed the tumor cell growth rate significantly increased after ectopic expression of SIX1 (Figure 2F) and decreased after silencing the endogenous SIX1 expression (Figure 3D), which were in accord with the findings from cell growth curve (Figures 2G, 3E). Transfecting the exogenous *SIX1* gene into BCPAP cells can increase both colony number and size (Figure 2H), and SIX1 knockdown otherwise resulted in approximately 50% colony growth inhibition in the TPC-1 cells (Figure 3F). Furthermore, cell migration and invasion were significantly increased in BCPAP cells with SIX1 overexpression (Figure 2I) and decreased by half in TPC-1 cells after knockdown of SIX1 (Figure 3G). These data suggested that SIX1 induced the oncogenic properties of PTC.

### SIX1 Protected PTC Cells Against H_2_O_2_-Induced Apoptosi*s*

**Figure 4:**
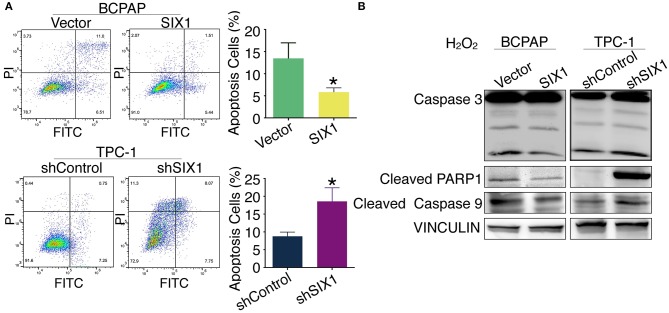
SIX1 attenuated H_2_O_2_-induced apoptosis in PTC cell lines. PTC cell lines were incubated for 24 h, and then exposed to 200 μM H_2_O_2_ for 24 h. Annexin V-FITC/PI assay was used to evaluate the apoptosis. Representative image of apoptosis assay and quantitative analysis (*n* = 3) **(A)**. The protein expression of caspase 3, cleaved PARP1 and cleaved caspase 9 in PTC cell lines was detected by Western blotting **(B)**. ^*^p < 0.05.

Increased proliferation and reduced apoptosis are the fundamental feature of cancer cells. Next, we evaluated whether SIX1 protected PTC cell lines from apoptosis assessed by Annexin V-FITC/PI staining. Flow cytometric analysis results indicated that H_2_O_2_ induced apoptosis in PTC cell lines. The proportion of apoptotic cells in BCPAP-SIX1 group was significantly reduced from 13.46 to 5.82% in comparison with BCPAP-Vector (*p* = 0.022) (Figure 4A). The proportion of apoptotic cells in TPC1-shSIX1 group was increased significantly from 8.75 to 18.56% in comparison with TPC1-shControl (*p* = 0.0133) (Figure 4A). The protein expression levels of caspase 3, cleaved PARP1 and cleaved caspase 9 were detected by Western blotting. The results showed that SIX1 significantly attenuated H_2_O_2_-induced upregulation of cleaved Caspase 3, cleaved PARP1 and cleaved Caspase 9 protein expression (Figure 4B).

### STAT3 Signal Is Responsible for the SIX1-Induced Proliferation of PTC Cells in an EYA1-Dependent Manner

**Figure 5:**
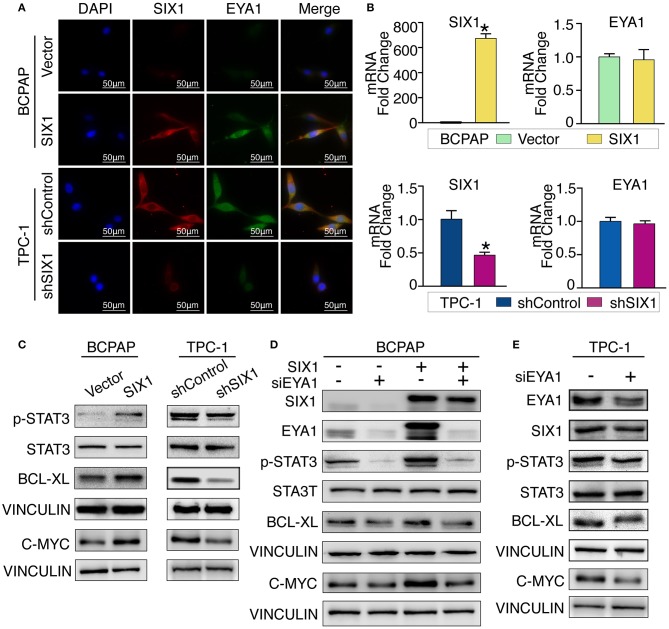
SIX1 activated STAT3 signaling pathway to promote PTC cells proliferation via EYA1. Immunofluorescence show the co-localization of SIX1 (red) and EYA1 (green) **(A)**. The relative mRNA level of SIX1 and EYA1 in PTC cell lines with SIX1 overexpression or silencing **(B)**. Western blot analysis of indicated protein from PTC cells with SIX1 overexpression or silencing **(C)**. Western blot analysis of indicated protein from BCPAP cells with SIX1 and/or EYA1 silencing **(D)**. Western blot analysis of indicated protein from TPC-1 cells with EYA1 silencing **(E)**. ^*^p < 0.05.

Although there is a consensus that EYA functions as a co-activator in SIX1-associated malignant properties, little is known about whether and how SIX1 modulates EYA1 expression. Immunofluorescence assay showed the positive relationship and co-localization of SIX1 and EYA1 (Figure 5A). Moreover, we carried out qRT-PCR to examine the mRNA change. However, the mRNA of *EYA1* did not change when *SIX1* was overexpressed in BCPAP or *SIX1* was knocked down in TPC-1 cells (Figure 5B), indicating that SIX1 induces the protein abundance of EYA1 at post-transcriptional level, rather than directly involves in the transcriptional regulation of EYA1 mRNA.

Cell cycle proteins regulated by SIX1 might be responsible for the proliferation in PTC. To address the underlying mechanisms, cell cycle elements were screened by western blot. SIX1 significantly promoted the abundance of C-MYC (Figure 5C). Since C-MYC is the downstream target of STAT3 signaling and other group has reported SIX1 can induce p-STAT3 expression in human keratinocytes (24), we hypothesized that SIX1 might promote C-MYC abundance via activation of STAT3 signaling. Ectopic expression of SIX1 could significantly upregulate the expression of p-STAT3 (Try705) as well as its downstream target BCL-XL. As expected, knockdown SIX1 in TPC-1 cell showed the opposite (Figure 5C).

To further determine whether EYA1 was required for the SIX1-induced STAT3 signaling activation. siRNA was used to transiently knock down EYA1 in BCPAP-vector and BCPAP-SIX1 cells. Intriguingly, the upregulation of p-STAT3, C-MYC, and BCL-XL induced by SIX1 was abolished after silencing EYA1. Additionally, knocking down EYA1 in BCPAP-Vector cells diminished endogenous expression of STAT3 signal and its downstream targets (Figure 5D). We evaluated the effects of EYA1 knock-down in TPC-1 cells which had higher endogenous expression of SIX1. The expressions of SIX1, p-STAT3, C-MYC, and BCL-XL abundance were down regulated after silencing EYA1 by siRNA in TPC-1 cells (Figure 5E).

**Figure 6:**
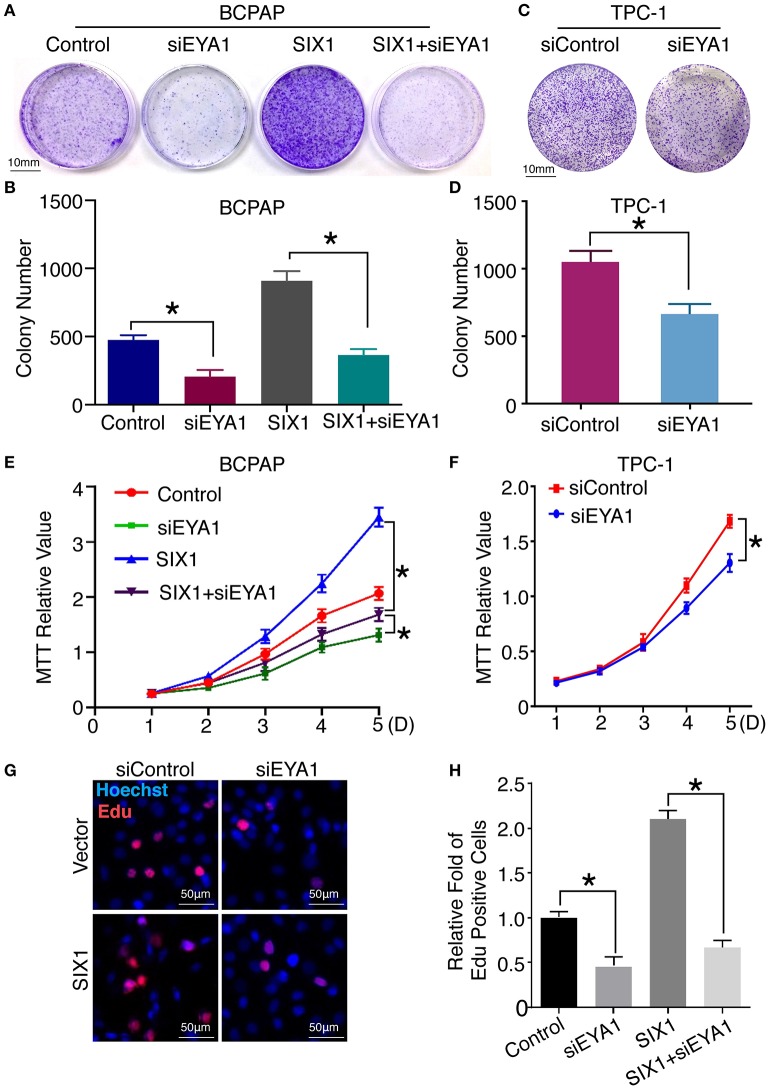
Interaction of SIX1 and EYA1 determined PTC cells proliferation. Representative image of colony formation from BCPAP cells with SIX1 overexpression and/or siEYA1 **(A)** and quantitative analysis (*n* = 3) **(B)**. Representative image of colony formation from TPC-1 cells with siEYA1 **(C)** and quantitative analysis (*n* = 3) **(D)**. MTT assay of BCPAP cells with SIX1 overexpression and/or siEYA1 (*n* = 5) **(E)**. MTT assay of TPC-1 cells with siEYA1(*n* = 5) **(F)**. Representative images of EdU assay of BCPAP cells with SIX1 overexpression and/or siEYA1 **(G)** and quantitative analysis (*n* = 3) **(H)**. ^*^p < 0.05.

Next, we compared the functional significance of SIX1/EYA1 interaction. Both colony formation assay and MTT showed that the proliferation ability was significantly reduced when EYA1 was knocked down in SIX1-overexpressing BCPAP cells (Figures 6A,B,E). In agreement, SIX1-induced DNA synthesis was inhibited by knock down EYA1, as evaluated by EdU incorporation assay (Figures 6G,H). On the other side, colony formation assay and MTT demonstrated that knockdown endogenous EYA1 can inhibit TPC-1 cell growth (Figures 6C,D,F). Together, these data suggest that the activation of STAT3 signaling induced by SIX1 is dependent on the presence of EYA1 in PTC cells.

### Silencing SIX1 Suppressed Tumor Growth as Well as STAT3 Signal Activation *in vivo*

**Figure 7:**
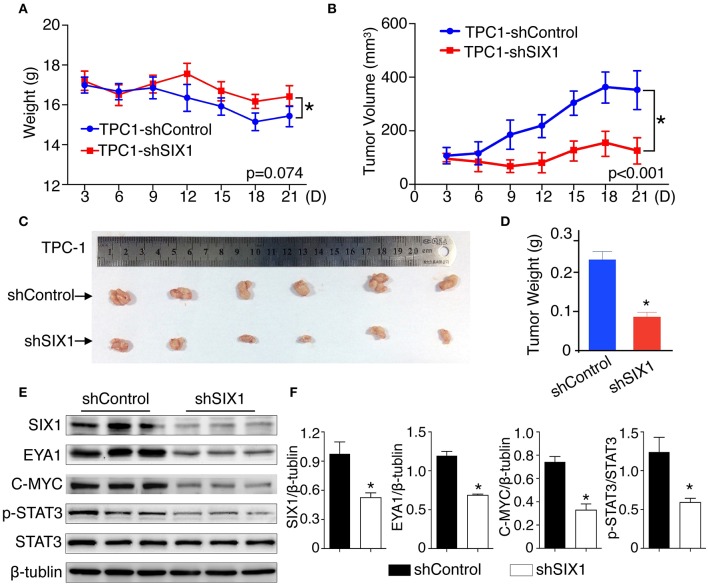
Silencing SIX1 suppressed tumor growth as well as STAT3 signal *in vivo*. TPC-1 cells with SIX1 silencing or vector control were implanted into immunodeficiency mice, the body weight **(A)** and tumor volume **(B)** was monitored (*n* = 20). Representative tumor image **(C)** and quantitative analysis (*n* = 20) **(D)** were showed. Western blot analysis of total protein from tumor tissue **(E)** and relative quantitative analysis (*n* = 3) **(F)**. ^*^p < 0.05.

To test whether blocking the expression of SIX1 abolished the PTC cell proliferation *in vivo*, 2 × 10^6^ TPC1-shControl or TPC1-shSIX1 cells were injected, respectively, into the subcutaneous fat of immunodeficient mice. Downregulation of the endogenous SIX1 by shRNA in TPC-1 cells not only showed a trend of slowing down the tumor associated weight loss (Figure 7A), but also significantly reduced the volume as well as the weight of xenografted tumors (Figures 7B–D). At the time of sacrifice, xenograft tumors were harvested and examined by western blot. Consistent with these findings *in vitro*, the tumors in TPC1-shSIX1 group retained SIX1 lower-expression *in vivo* and the levels of EYA1, p-STAT3 (Try705) and C-MYC were significantly decreased compared with shControl group (Figures 7E,F). Taken together, these results highlight the potential for targeting SIX1 in the comprehensive therapy for PTC patients.

## Discussion

Exciting outcome of molecular targeting therapy is driving the need for better understanding the tumor biological features of each patient (7). Recent evidence has linked RDGN family with the progression of various cancers, indicating it might be a potential therapeutic target for precision medicine (11–18, 25). In the present study, our results first showed that SIX1 protein elevated in thyroid malignant tumor. In papillary thyroid carcinoma, high expressions of SIX1 and EYA1 were associated with advanced age, lymph node metastasis and clinical stage. Functional assays suggested SIX1 not only enhanced PTC proliferation, but also provided significant protection against apoptosis, which relied on the activation of STAT3 signaling. The analysis of patients' samples indicated EYA1 was positively correlated with SIX1. Not only the ectopic expression of SIX1 increased EYA1, but also STAT3 signaling activation induced by SIX1 depended on EYA1.

Although Six1 had intrinsic transcriptional domain, its activation required Eya family to be the co-activator (26). Co-transfection of Six/*so* and Eya/*eya* induced a huge synergistic activation of the downstream targets, whereas Six or Eya alone showed a low level of transcription. Therefore, it generally recognized that Six1-Eya function as a transcriptional complex (11). However, the coordinated mechanism between SIX1 and EYAs in human is largely elusive. Current studies are mainly focused on the functional association between SIX1 and EYAs. It has been reported SIX1 bound to EYA by a single amphipathic helical structure, an essential part for SIX1-associated metastatic phenotypes (27). One significant finding in our study was that SIX1 induced EYA1 protein expression in PTC and modulated its abundance at the post-transcriptional level. Recent evidence indicated that cyclin-dependent kinase 6 (CDK6) bound to and promoted degradation of the EYA2 protein, and SIX1 partially stabilized EYA2 against CDK6 induced degradation (28), which is in support of our finding. Our results also showed EYA1 was necessary for STAT3 signaling activation as well as the cellular proliferation induced by SIX1. Based on these data, it indicates SIX1 might stabilize the EYA1 protein rather than activate the gene transcription directly, suggesting a reciprocal regulation between SIX1 and EYA family.

Another intriguing finding is STAT3 signaling is involved in the regulation of SIX1 in PTC. This is consistent with a previous report demonstrating that SIX1 can increase the phosphorylation of STAT3 in HPV16-immortalized human keratinocytes (24). Given the role of STAT3 signaling in regulating cell proliferation, vascular formation and immune response associated with cancer progression, it is important to explore novel targets that inhibit the ectopic activation of STAT3 signaling (29). By activating pro-proliferative and pro-survival genes, including C-MYC and Cyclin D1, and anti-apoptotic BCL-2 or BCL-XL, STAT3 mediated tumorigenesis by protecting cells from apoptotic stress, and promoting cell-cycle progression and inflammation response in multiple cancers (30). STAT3 phosphorylated on Try705 in the carboxyl-terminal transactivation domain is regarded as the key to amplify STAT3 signal. In thyroid, STAT3 pathway was found to be involved in the onset and invasion of tumor (31). STAT3 interacted with the sonic hedgehog (SHH) pathway, which can predict the prognosis or guiding the personal therapy against PTC (32). Ekpe-Adewuy et al. revealed that growth factor receptor-alpha (PDGFRα) promoted EMT of PTC cells via provoking STAT3 signal pathway (33). Our study found SIX1 activated STAT3 signaling in an EYA1-dependent manner by increased the level of phospho-Try705. *In vitro* experiments indicated that SIX1 increased the phosphorylation of STAT3, leading to an increasing of C-MYC and BCL-XL to promote cell proliferation and anti-apoptosis. In addition, the activation of STAT3 signaling induced by SIX1 relied on EYA1. Silencing EYA1 expression abolished the SIX1-medicated STAT3 signaling activation in cell proliferation. Our mouse xenograft model also confirmed that decreased SIX1 abundance was associated with significantly reduced tumor volume and suppression of STAT3 signaling. These findings suggest that STAT3 signaling plays a crucial role in the functional significance of SIX1 in thyroid cancer, and it may raise a possibility that JAK/STAT3 inhibitors can be treated in those PTC patients with high expression of SIX1.

Our study indicated that SIX1 and EYA1 might be used as malignancy hallmarks in papillary thyroid cancer. Higher levels of SIX1 and EYA1 were found correlated with advanced age and lymph node metastasis, which are well-known poor prognostic factors in PTC. Although the tumor size showed no significant relationship with SIX1 or EYA1 expression, the functional assays identified that overexpression of SIX1 contribute to the proliferation of PTC cells, whereas knockdown SIX1 showed the opposite. Moreover, the success of lipid nanoparticles (LNP)-formulated siRNA targeting VEGF and kinesin spindle protein (KSP) in cancer patients with liver metastasis raised a hope that silencing SIX1 expression using shRNA or miRNA maybe a potential cancer treatment strategy (34). For example, microRNA-185 translationally represses SIX1 and thereby sensitizes SIX1-overexpressing cancer cells to TRAIL-induced apoptosis (35). It is known that EYAs mediate the transcriptional activation of SIX1 (27). Our study also showed that EYA1 is responsible for SIX1-induced STAT3 signaling activation, indicating disruption of EYA1 function may suppress the SIX1-mediate tumor growth in PTC. To date, Benzbromarone and its derivative, Benzarone were also found to be well-recognized EYA inhibitors (36). The major metabolite of Benzbromarone, 6-hydroxy Benzbromarone, is a more powerful inhibitor of EYAs, and may block tumor growth by inhibiting angiogenesis (37). Therefore, a combination treatment with either SIX1 or EYA1 inhibitors may provide benefit in a properly selected group of PTC patients.

## Data Availability Statement

All datasets generated for this study are included in the article/Supplementary Material.

## Ethics Statement

This study was approved by the Institutional Review Board of Tongji Hospital.

## Author Contributions

GW and KWu developed the hypothesis and designed the experiments. DK, AL, YL, QC, KWa, DZ, JT, and YD performed the experiments. DK prepared the manuscript. ZL contributed to the pathology review and interpretation.

### Conflict of Interest

The authors declare that the research was conducted in the absence of any commercial or financial relationships that could be construed as a potential conflict of interest.

## Supplementary Material

Supplementary Data Sheet 1 STR profile of BCPAP.

Supplementary Image 1 STR profile of TPC-1.
