# Supplementary material for: SIX1 Activates STAT3 Signaling to Promote the Proliferation of Thyroid Carcinoma via EYA1
Source: Front Oncol. 2019 Dec 20;9:1450. doi: 10.3389/fonc.2019.01450 (PMC6933607; doi:10.3389/fonc.2019.01450)

Applied Biosystems

GeneMapper 4.0

# Cell Line Authentication-8

| Sample File | Sample Name | Panel | OS | SQ |
| --- | --- | --- | --- | --- |
| 77_E10_CellLineAuthentication-0104.FSA | BCPAP | STR Profile 1-human | | |

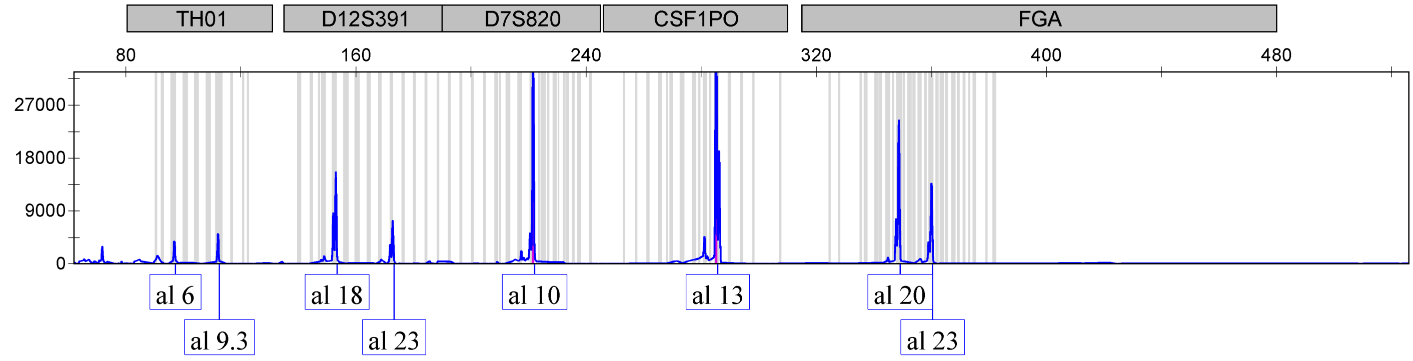


| Sample File | Sample Name | Panel | OS | SQ |
| --- | --- | --- | --- | --- |
| 49_A07_CellLineAuthentication-0103.fsa | BCPAP | STR Profile 2-human | | |

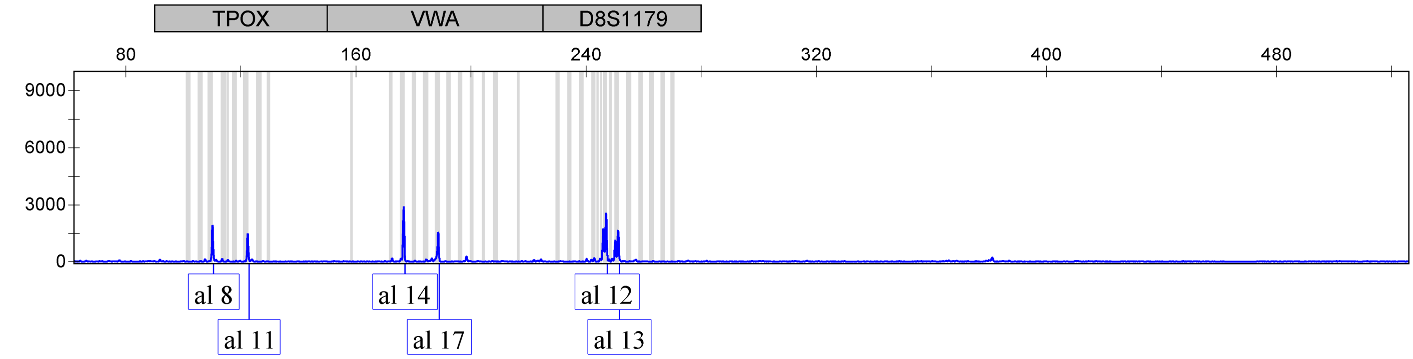


| Sample File | Sample Name | Panel | OS | SQ |
| --- | --- | --- | --- | --- |
| 90_B12_BDLYBu(NOS1-4)1-7(DAT1-4)15-0105.fsa | BCPAP | STR Profile 3-human | | |

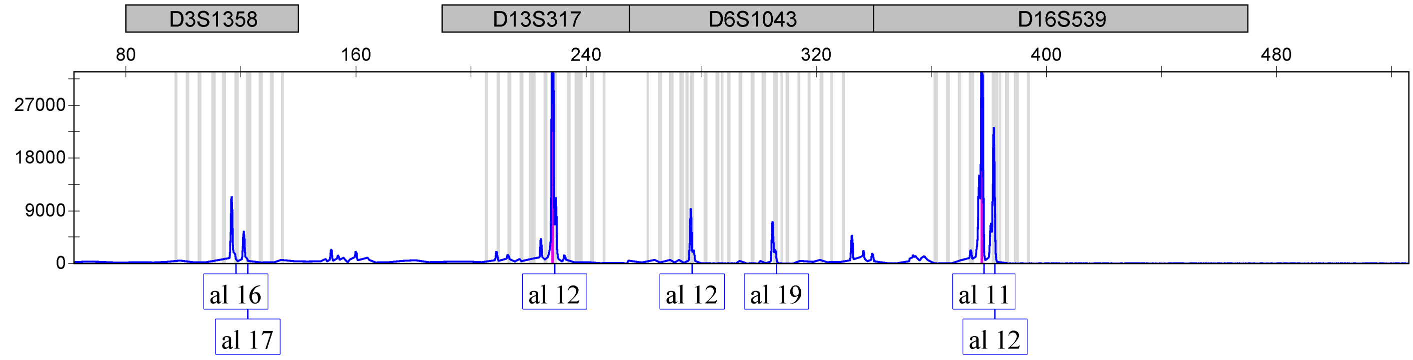

| Sample File | Sample Name | Panel | OS | SQ |
|---|---|---|---|---|
| 41_A06_CellLineAuthentication-0104.FSA | BCPAP | STR Profile 4-human | | |

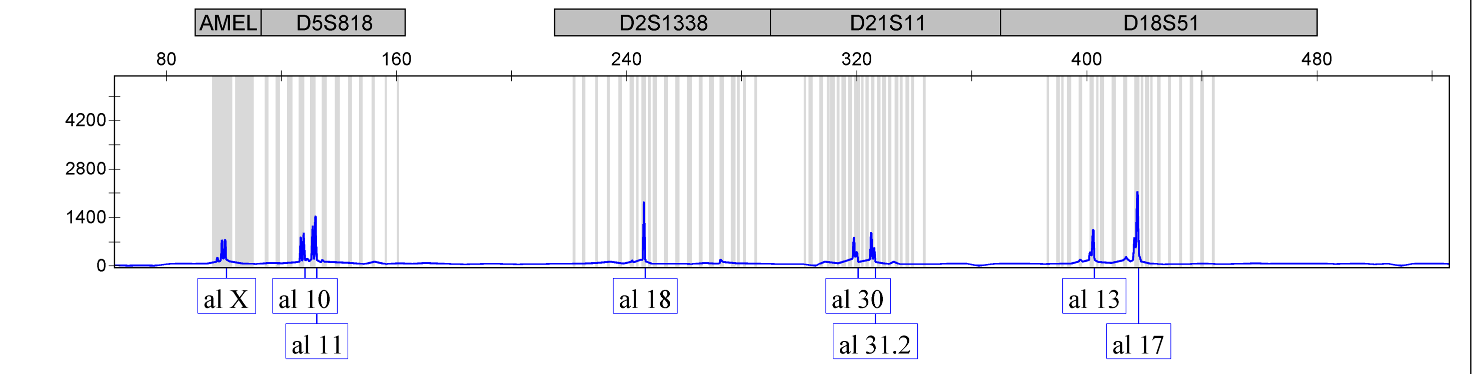

Applied Biosystems
GeneMapper 4.0

# Cell Line Authentication-8

| Sample File | Sample Name | Panel | OS | SQ |
|---|---|---|---|---|
| 17_A03_CellLineAuthentication-0103.fsa | BCPAP | STR Profile 1-human | | |

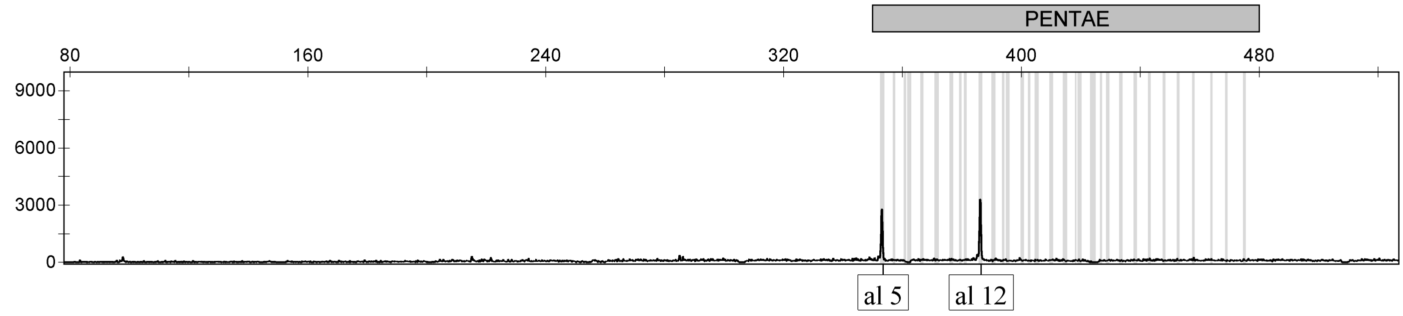


| Sample File | Sample Name | Panel | OS | SQ |
|---|---|---|---|---|
| 49_A07_CellLineAuthentication-0103.fsa | BCPAP | STR Profile 2-human | | |

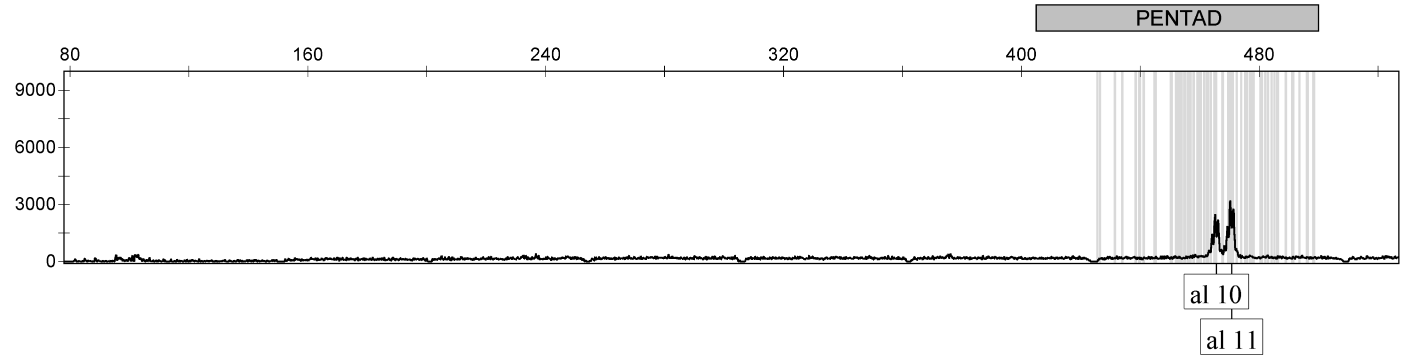


| Sample File | Sample Name | Panel | OS | SQ |
|---|---|---|---|---|
| 09_A02_CellLineAuthentication-0104.FSA | BCPAP | STR Profile 3-human | | |

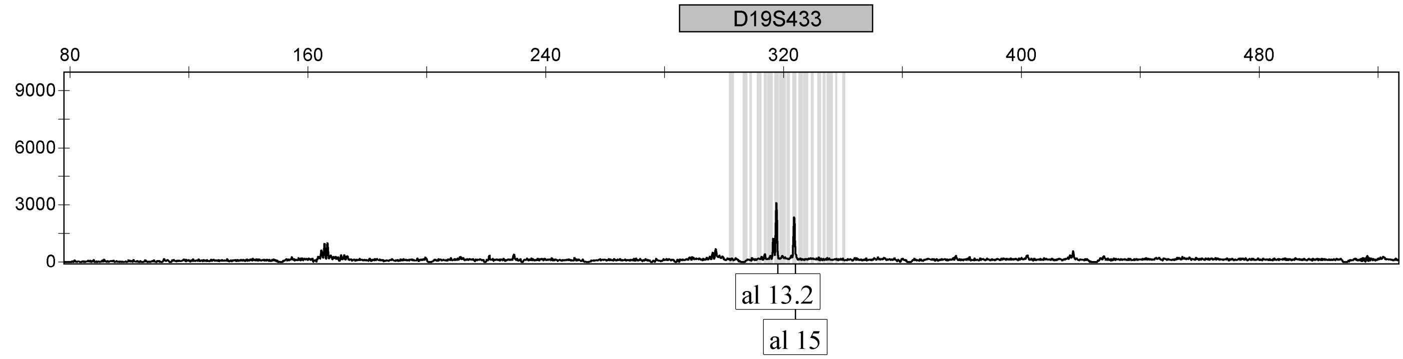

Supplement: Supplementary Data Sheet 1 — STR profile of BCPAP. [file Data_Sheet_1.PDF]
