# Supplementary figures and images for: SIX1 Activates STAT3 Signaling to Promote the Proliferation of Thyroid Carcinoma via EYA1
Source: Front Oncol. 2019 Dec 20;9:1450. doi: 10.3389/fonc.2019.01450 (PMC6933607; doi:10.3389/fonc.2019.01450)

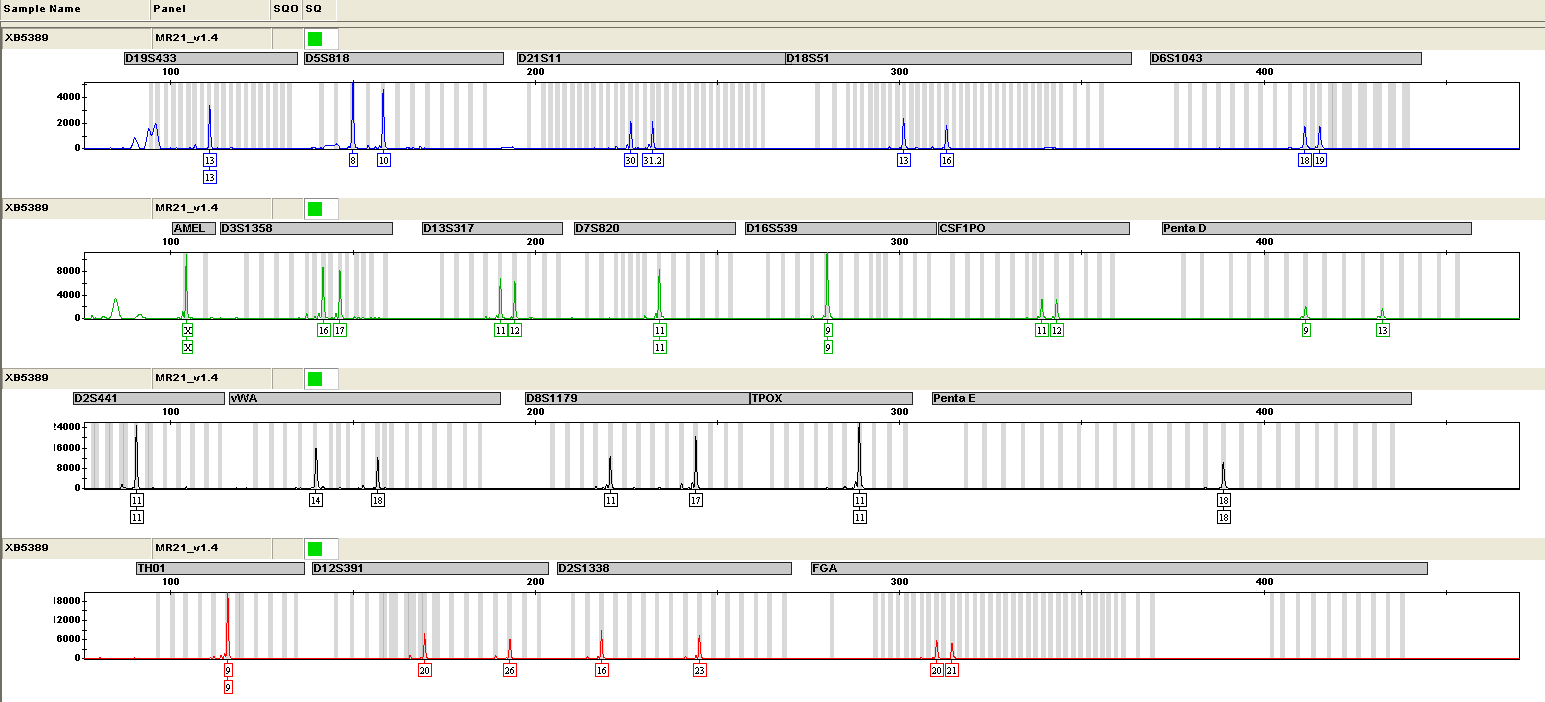

Supplement: Supplementary Image 1 — STR profile of TPC-1. [file Image_1.PNG]
